# Time-Dependent Endocrine Signaling as a Modifiable Therapeutic Variable in Advanced Prostate Cancer

**DOI:** 10.5152/tud.2026.25129

**Published:** 2026-04-10

**Authors:** Ivan Bivolarski

**Affiliations:** Oncology Centre in Burgas LTD, Burgas, Bulgaria

Dear Editor,

Prostate cancer remains one of the most therapeutically challenging malignancies in male oncology, largely due to the development of resistance to androgen deprivation therapy (ADT) and next-generation androgen receptor signaling inhibitors (ARSi), including abiraterone, apalutamide, darolutamide, and enzalutamide. While substantial progress has been made in elucidating molecular drivers of therapeutic escape, the prevailing clinical paradigm continues to treat ADT and AR-targeted agents as time-independent interventions, implicitly assuming uniform pharmacodynamic efficiency regardless of the biological timing of administration.^1^

This perspective challenges that assumption. Emerging evidence from endocrine physiology, circadian biology, and translational oncology suggests that time of day represents a modifiable therapeutic variable capable of influencing hormone receptor dynamics, steroid metabolism, immune tone, and treatment response. In prostate cancer specifically, androgen secretion, glucocorticoid rhythms, androgen receptor transcriptional activity, and related molecular pathways exhibit circadian oscillations regulated by central and peripheral molecular clocks. Experimental and preclinical data further indicate that receptor cofactors and downstream transcriptional programs may display time-dependent variability, supporting biological plausibility for temporal modulation of androgen signaling. However, current clinical protocols do not consider whether aligning treatment delivery with these rhythms may enhance therapeutic effectiveness or delay the emergence of resistance.^2^^,^^3^

It should be explicitly acknowledged that most clinical evidence supporting chronotherapy originates from non-urologic malignancies, including chemotherapy, radiotherapy, and immunotherapy studies. Direct prostate cancer–specific chrono-interventional data remain limited.

Nevertheless, these findings raise important translational questions within the context of endocrine-driven malignancies:

Could synchronizing ADT or ARSi administration with endocrine circadian phases improve therapeutic response?
Could circadian misalignment accelerate resistance and disease progression?

Mechanistic plausibility is supported across multiple biological domains. Cortisol and testosterone rhythms, gonadotropin signaling, steroidogenesis, and androgen receptor transcription demonstrate time-regulated dynamics, suggesting that pharmacologic interventions applied during suboptimal circadian phases may encounter altered receptor sensitivity, modified drug metabolism, or relative immune suppression. Conversely, treatment delivered during biologically favorable windows could theoretically enhance apoptotic signaling, delay clonal adaptation, or improve synergy with host immune surveillance.

This hypothesis aligns with emerging evolutionary models of tumor adaptability, in which therapeutic pressure applied during predictable or biologically vulnerable phases may influence cancer evolutionary trajectories. If endocrine oscillations regulate elements of the tumor microenvironment or transiently alter receptor configuration, therapeutic timing could become a tool to shape tumor evolution rather than merely react to resistance.

To explore this hypothesis, a pragmatic pilot study design could include:

1. Standardized timing of daily oral androgen receptor pathway inhibitors.
2. Integration of serial endocrine and inflammatory biomarkers, including exploratory markers such as neutrophil-to-lymphocyte ratio, alongside more contemporary indicators such as AR-V7 status, circulating tumor DNA dynamics, or transcriptional circadian signatures.
3. Correlation with clinical endpoints, including prostate-specific antigen (PSA) kinetics, progression-free survival, toxicity profiles, and quality of life.
4. Consideration of key confounders, including sleep disorders, corticosteroid use, obesity, and shift work.

Feasibility and clinical implementation warrant careful consideration. Timing adherence may be realistic for daily oral therapies but less so for depot formulations. Circadian variation in drug pharmacokinetics and toxicity remains incompletely characterized for ARPIs, underscoring the exploratory nature of such studies. Importantly, current timing practices in large randomized trials have been largely pragmatic rather than chronobiologically driven. For example, in the Alliance A031201 trial, enzalutamide, abiraterone, and prednisone were administered at fixed times primarily based on tolerability and pharmacokinetic considerations, without evidence of an overall survival benefit attributable to timing itself.^4^ This observation highlights the absence of chronobiologically designed trials rather than disproving a potential chrono-effect.

The objective of this Letter is not to assert definitive conclusions, but to encourage scientific dialogue and stimulate translational and clinical collaboration around chrono-informed endocrine therapy. Defining time as a modifiable therapeutic variable represents a realistic and testable next step in advanced prostate cancer research. If validated, time-synchronized ARSi administration could represent a low-cost, widely applicable strategy to optimize outcomes for patients with advanced prostate cancer.
